# 

**Published:** 1883-01

**Authors:** 


PAGE

ORIGINAL COMMUNICATIONS.—Latent Glanders in the Horse. G. W. Bololer, M.D.... ...... 1
Are there Pysiological Affinities in the Blood. Allen S. Heath, M.D ....................... 8
Physiical Diagnosis. E. Benjamin Ramsdell, M.D ............................................ 11
Causes of Change in Animal Forms. Hubbard W. Mitchell, M.D.................................. 19
Some Diseases of the Eye in Lower Animals. William Oliver Moore, M.D.......................... 31
Atavism and Reproduction. John W. Navin, V.S. ... .............................................. 36
Early History of Veterinary Medicine in the United States. R. Jennings, V.S ............. 42

EDITORIAL.—Veterinary Societies—English Veterinary Colleges and their Graduates—Attempts at Veterinary Colleges—Legal Status of Veterinary Colleges in New York City—Stomach Digestion in the Horse—Sexual Mania in Elephants—Goats and Homœopathy—Domestic Pets and Skin Diseases—The Action of Pilocarpin upon Horses—Cattle—Notes.................................. 50-58

CASE DEPARTMENT—Severe case of Spongy Iritis in a Horse—Adenoma of the Breast in a Dog—Ideopathic Tetanus—Treatment of Chronic Catarrh by Carbolic Acid without Trepanning....... 59-63

CORRESPONDENCE.......................................................................... 64

OBITUARY................................................................................ 67

REVIEWS.—Anatomical Tochnolegy as Applied to the Domestic Cat—Bovine Medicine and Surgery—Animal Plagues—Stable Management—Annual Report Veterinary Department—Classification Muscles of the Horse—Books and Pamphlets Received........................................ 68-72

PROGRESS OF VETERINARY SCIENCE.—Heredity—Diseased Fowls—Bronchitis in Birds—A Good Medicine for Poultry—A Bacellus, or a Fat Crystal—The Pepsin of Sheep—Birds and Trichinæ—Hydrobromate of Homatropine—A Striking Case of Elephantasis in a Horse—Softening of Bones in a Cow—The Horse Family—Astragalus Mollissimus—Parasitic Disease in a Dolphin—Diseases and Accidents Among Ostriches—Nervous Diseases of Pachyderms—Cranial Capacity of the Horse—Incubation of the Rabies in the Dog—Recovery from Rabies—A Case of Hydrophobia Treated Successfully with Aconite—Interesting to Veterinary Dissectors—Flexible Anatomical Preparation of Joints—Intestinal Disorders in Horses—The Influence of Animal Food on the Production of Wool—Comparative Dietetics—Induced predisposition to Anthrax—English Veterinary News.......................................................................... 73-80

NEWS AND MISCELLANY.—Tsetse Fly—Ostrich Breeding—Ration of Hay for a Horse—Daily Yield of Goats' Milk—Donation to Veterinary College—Berlin Veterinary Medical Schools—North-western Veterinary College—Philadelphia Veterinarians and Dishonest Druggists—Blood-poisoning—Poisoning by Arsenic—Cats' Attempt at Suicide—What is Cruelty to a Cat—Overfed Pigs—A Cow as is a Cow—Parasites in Stock—International Veterinary Congress—Cattle Quarantine Stations—Exports and Imports of Neat Cattle—Legislation Regarding Neat Cattle—Typhoid Fever Among Live Stock—Specimen Veterinarian............................................ 81-85

